# Long-term Use of Demeclocycline for the Treatment of Chronic Hyponatremia

**DOI:** 10.7759/cureus.6415

**Published:** 2019-12-18

**Authors:** Amardeep Singh, Bhagwan Dass, Abutaleb Ejaz, Atul Bali

**Affiliations:** 1 Nephrology, University of Florida Health, Gainesville, USA; 2 Nephrology, University of Virginia, Farmville, USA

**Keywords:** demeclocycline, hyponatremia, chronic hyponatremia, v2-receptor, siadh, vasopressin, vasopressin-2 receptor antagonist, euvolemia, tolvaptan

## Abstract

Syndrome of inappropriate antidiuretic hormone secretion may be a frequent accompaniment of aging without an obvious central nervous system or pulmonary disorder, tumor, or drugs as the confounding factor. Treatment is often warranted due to complex symptomatology that is often associated with unfavorable clinical outcomes. Although V_2_-receptor antagonists are effective in increasing serum sodium, their side-effect profile and cost may be a barrier to its use. We report a patient with symptomatic, severe, chronic hyponatremia requiring multiple hospitalizations, who was successfully maintained on long-term demeclocycline therapy.

## Introduction

Hyponatremia is a vexing clinical problem associated with an increased risk for mortality. The pathomechanisms of hyponatremia are varied, and treatment options include fluid restriction, isotonic and hypertonic solutions, and diuretics. Tolvaptan, an oral vasopressin 2 (V2) receptor antagonist, was effective in increasing serum sodium (SeNa) concentrations in the short (30 days) and long (2 years) term in hyponatremic patients [1,2]. However, concerns regarding liver dysfunction and costs are limitations to its use. Demeclocycline has also been used to treat hyponatremia; however, its dose adjustments can be complex and its use in clinical practice is not well defined. We report a patient with symptomatic, severe, chronic hyponatremia requiring multiple hospitalizations, who was successfully maintained on long-term demeclocycline therapy.

## Case presentation

A 76-year-old male with past medical history of hypertension, diabetes, chronic obstructive pulmonary disease, coronary artery disease, and major depressive disorder was admitted with two to three days of altered mental status and generalized weakness in the setting of recurrent episodes of hyponatremia (SeNa, 109-115 meq/L) requiring three hospitalizations in the previous three months. On each admission, he was treated with fluid restrictions, 3% normal saline (NS) with transient improvement in his SeNa to 125-130 mEq/L. He was not on diuretics, denied any nausea, vomiting, or pain. Current physical examination revealed blood pressure 124/54 mmHg, heart rate 65 bpm, respiratory rate 16/min, body weight 108.2 kg; cardiovascular, respiratory, gastrointestinal, genitourinary, musculoskeletal, and neurological examination was unremarkable; and there was no lower extremity edema. 

Chest x-ray revealed cardiomegaly without circulatory congestion. EKG showed normal sinus rhythm, normal PR, QRS, and QT intervals. Laboratory values: SeNa 109 mEq/L (normal, 135-145 mEq/L), potassium 3.2 mEq/L (normal, 3.5 to 5.5 mEq/L), chloride 76 mEq/L (normal, 96 to 106 mEq/L), total bicarbonate 20 mEq/L (normal, 23 to 30 mEq/L), blood urea nitrogen 12 mg/dL (normal, 7 to 20 mg/dL), creatinine 0.6 mg/dL (normal, 0.6 to 1.2 mg/dL), calcium 8.8 mg/dL (normal, 8.5 to 10.5 mg/dL), uric acid 2.1 mg/dl (normal, 3.4-7.0 mg/dL), serum osmolality 241 mOsm/L (normal, 285-295 mOsm/kg), and urine osmolality 443 mOsm/L. Urine-specific gravity 1.025, random urine sodium 41 meq/L, urine creatinine 65.2 mg/dL, and fractional excretion of sodium 0.4%. Head CT was negative.

His home medications (citalopram and lisinopril) were discontinued. Lantus insulin and metformin were continued. Symptomatic hyponatremia was treated with 3% NS at 50 mL/hour with a goal to correct SeNa by 0.5-1.0 meq/L/hour and not to exceed by 6 meq/L in six to eight hours, and to stop at moderation of symptoms or the achievement of a SeNa concentration of 125 to 130 mmol/L. His SeNa was 129 mEq/L on day 3 but then worsened again to 124 meq/L. The patient met the criteria for syndrome of inappropriate antidiuretic hormone (SIADH) based on hyponatremia, serum hypo-osmolality, urine osmolality > 100 mOsmol/kg, urine sodium > 40 mEq/L, normal potassium, and no acid base problems. Subsequent work-up failed to unearth any malignancy. He was initiated on demeclocycline at 300 mg twice daily.

**Figure 1:**
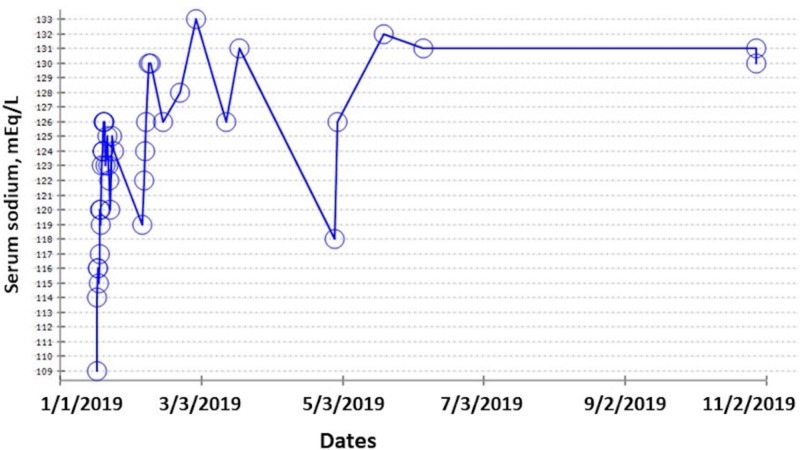
Clinical course of improvement in serum sodium with demeclocycline.

Figure 1 demonstrates the clinical course over the next nine months. SeNa improved and remained relatively stable, his weight decreased significantly, his overall sense of well-being improved, and he did not require any further hospitalizations.

## Discussion

SIADH is the most common cause of euvolemic hyponatremia, which is characterized by non-osmotic release of vasopressin leading to increased water permeability in the renal collecting duct, water retention, and increased circulatory blood volume. Our patient did not have clinical evidence for hypervolemia. Although the etiology of SIADH includes central nervous system and pulmonary disorders, tumors, and drugs, it can be frequently associated with aging and is a predictor of poor outcomes [3]. The etiology of SIADH in this patient remains elusive, but treatment to prevent hemodynamic, neuromuscular, and cognitive impairments was warranted.

Vasopressin increases water permeability of the collecting duct principal cells by stimulating vasopressin V2 receptor in the basolateral plasma membrane. This activates G proteins, which stimulate adenylyl cyclase, resulting in increased intracellular cyclic adenosine monophosphate concentration and activation of protein kinase A. The exact mechanism of demeclocycline-related attenuation of hyponatremia is unknown; however, it has been shown to reduce aquaporin-2 (AQP-2) expression in the renal inner medulla [4]. Improvement in hyponatremia in the patient was associated with significant weight loss (water diuresis) without worsening of renal function suggesting the influence on AQPs.

Phosphate diabetes, i.e., inability to reabsorb tubular phosphate and increase in blood urea nitrogen, has been reported with the use of demeclocycline dose > 1,200 mg/day [5]. These complications were not observed in this patient in whom the demeclocycline dose did not exceed 600 mg/day. His liver function tests remained normal.

## Conclusions

We have demonstrated long-term successful management of hyponatremia associated with SIADH with demeclocycline. Our experience suggests that demeclocycline may be an alternate option in patients with difficult-to-manage hyponatremia associated with SIADH of unclear etiology.
